# Inflammatory markers and increased risk of chronic kidney disease in patients with coronary artery disease: findings from a multicenter cohort study

**DOI:** 10.3389/fendo.2026.1827326

**Published:** 2026-04-23

**Authors:** Yan Yu, Zhenbao Wang, Danyang Yu, Keli Li, Meng Chen, Haina Li, Ce Yuan

**Affiliations:** 1 Department of Cardiology II and Geriatric Management Ward, Zhangye Second People’s Hospital, Zhangye, China; 2 Department of Cardiothoracic and Vascular Surgery, Qingdao Municipal Hospital, University of Health and Rehabilitation Sciences, Qingdao, China; 3 Department of Ophthalmology, University of Health and Rehabilitation Sciences (Qingdao Central Hospital), Qingdao, China; 4 Department of General Medicine of Huangshan City People’s Hospital, Huangshan, China; 5 Suzhou Hospital of Anhui Medical University, Suzhou, China; 6 Zhejiang Xiaoshan Hospital, Hangzhou, China; 7 Department of Emergency Medicine, The Quzhou Affiliated Hospital of Wenzhou Medical University, Quzhou People’s Hospital, Quzhou, Zhejiang, China

**Keywords:** cardiorenal syndrome, chronic kidney disease, coronary artery disease, inflammatory markers, metabolic disturbances

## Abstract

**Background:**

Coronary artery disease (CAD) is associated with persistent inflammation, which plays an important role in the development of renal dysfunction. This study investigated the association between composite inflammatory indices and the long-term risk of chronic kidney disease (CKD) in patients with CAD, and further aimed to identify the inflammatory marker with the best predictive performance.

**Methods:**

We included 5181 patients with CAD from three centers. Multivariable-adjusted Cox regression models and cumulative risk curves (CRC) were used to evaluate the associations between four composite inflammatory markers—aggregate index of systemic inflammation (AISI), systemic inflammatory response index (SIRI), systemic immune-inflammation index (SII), and platelet-to-lymphocyte ratio (PLR)—and CKD risk. Restricted cubic splines (RCS) were applied to examine dose-response relationships, while predictive performance was compared using receiver operating characteristic (ROC) analysis, the C-index, and random forest (RF) variable importance.

**Results:**

Multivariable Cox regression showed that all four inflammatory markers were significantly associated with an increased risk of CKD, and these findings were further supported by CRC analysis. RCS analysis demonstrated clear dose-response relationships, with risk thresholds identified at AISI >115, SIRI >0.96, SII >458, and PLR >123. Among all markers, AISI showed the best predictive performance according to the combined evaluation of ROC analysis, the C-index, and RF variable importance.

**Conclusion:**

Elevated inflammatory marker levels were significantly associated with a higher risk of CKD in patients with CAD. Among the markers evaluated, AISI demonstrated the strongest predictive value and may serve as a useful indicator for early risk stratification and clinical management of CKD in this population.

## 1 Introduction

Chronic kidney disease (CKD) is a long-term impairment of kidney function characterized by reduced glomerular filtration rate and persistent proteinuria. It can progress to end-stage renal disease and lead to metabolic disturbances, electrolyte imbalance, and systemic complications involving the cardiovascular, skeletal, and hematologic systems (1, 2). Among patients with CKD, cardiovascular events remain the leading cause of death (1, 3–5). As populations continue to age, the prevalence of both CKD and cardiovascular disease has increased, and the coexistence of multiple chronic conditions further raises the risk of mortality (6–8). Coronary artery disease (CAD) frequently coexists with CKD and is associated with a greater risk of renal dysfunction, making patients with CAD a high-risk population for CKD. When these two conditions occur together, mortality increases substantially and quality of life declines markedly (9, 10). Therefore, early assessment and continuous monitoring of CKD risk should be prioritized in the management of CAD in order to prevent disease onset and progression and to improve long-term outcomes.

Traditionally, the development of CKD in patients with CAD has mainly been attributed to conventional risk factors such as poor blood pressure control, smoking, obesity, dyslipidemia, diabetes-related renal injury, and heart failure, whereas the contribution of persistent inflammation has received less attention (11–13). However, CAD may also impair renal function more directly through several interconnected mechanisms, including reduced renal perfusion secondary to heart failure, chronic inflammation, abnormal activation of the neurohormonal system, and cardiorenal syndrome, all of which contribute to progressive kidney injury and eventual CKD development (14–17). Recent studies have increasingly suggested that inflammation plays a central role not only in CAD itself but also in the systemic disorders associated with CAD, including kidney injury (18–20). Patients with CAD often remain in a chronic inflammatory state characterized by sustained release of inflammatory mediators (21, 22). Because the kidneys are particularly vulnerable to these inflammatory effects, they may undergo a range of pathological changes, such as endothelial injury, increased vascular pressure, plaque formation, and activation of the renin-angiotensin system (RAS), ultimately resulting in renal dysfunction (23–26). If this inflammatory state persists over time, renal injury may progressively worsen and thereby promote the onset and progression of CKD (26, 27).

Previous studies evaluating inflammatory status have often relied on single biomarkers, such as C-reactive protein, procalcitonin, lymphocyte count, or neutrophil count. However, these indicators have limited ability to comprehensively reflect the overall inflammatory state of the body (28, 29). In recent years, increasing attention has been given to novel inflammatory markers that can provide a more integrated assessment of immune and inflammatory status (28, 30, 31). These include the aggregate index of systemic inflammation (AISI), systemic inflammatory response index (SIRI), systemic immune-inflammation index (SII), and platelet-to-lymphocyte ratio (PLR) (30, 31). By combining multiple parameters derived from routine blood tests, these composite indices offer a more comprehensive measure of systemic inflammatory activation than single markers and have shown strong predictive value in a wide range of diseases (29–32). For example, SIRI has shown good performance in predicting stroke risk in patients with hypertension and has also demonstrated value in the assessment of bone health and osteoporosis (31). Likewise, AISI has shown superior predictive ability in the evaluation of fatty liver disease (30). In addition, these novel inflammatory markers have demonstrated strong prognostic value in severe conditions such as sepsis (28, 33).

Given the important role of inflammation in CAD and CAD-related renal injury, together with the promising predictive value of these emerging inflammatory markers across multiple diseases, this multicenter cohort study was designed to investigate the association between inflammatory markers and the future risk of CKD in patients with CAD. In addition, the study aimed to determine which marker performs best in evaluating inflammatory burden and predicting CKD risk in this population. It is expected that these findings will provide a more convenient and accurate tool for the early identification and intervention of CKD risk among patients with CAD.

## 2 Material and methods

### 2.1 Study population

**Figure 1:**
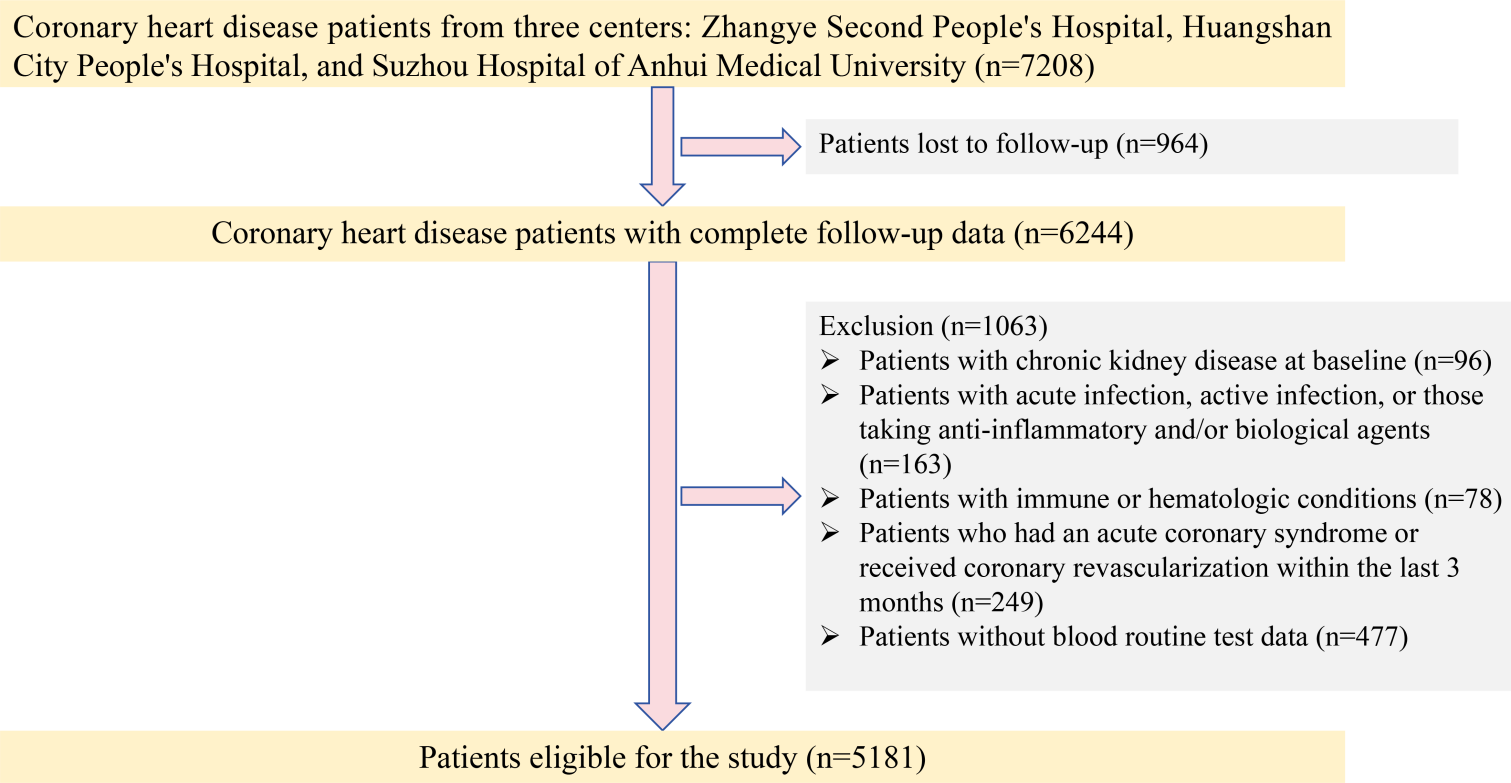
Screening process of the study population.

This study initially enrolled 7208 patients with CAD from three medical centers: the Second People’s Hospital of Zhangye City, Huangshi People’s Hospital, and Suzhou Hospital of Anhui Medical University. Of these, 6,244 patients completed follow-up. Patients were then excluded if they had CKD at baseline, had acute or active infections, were using anti-inflammatory and/or biological agents, had immune or hematologic disorders, or had experienced acute coronary syndrome or undergone coronary revascularization within the previous 3 months. After these exclusions, 5,181 patients with CAD were included in the final analysis. The detailed patient selection process is shown in **Figure 1**.

This study was conducted in accordance with the ethical principles of the Declaration of Helsinki. Ethical approval was obtained from the ethics committees of all three participating hospitals: Zhangye Second People’s Hospital (Approval No. ZYEY20191103), Huangshi People’s Hospital (Approval No. HS20210611), and Suzhou Hospital of Anhui Medical University (Approval No. SZ.N.20221109). Written informed consent was obtained from all participants before enrollment.

### 2.2 Data collection

Patient data were comprehensively obtained from electronic medical records, medical insurance databases, and follow-up records. For analysis, the collected variables were systematically grouped into three categories. First, demographic and clinical characteristics included sex, age, body mass index (BMI), systolic blood pressure, diastolic blood pressure, smoking status, and alcohol consumption. Second, laboratory parameters included complete blood count, alanine aminotransferase (ALT), aspartate aminotransferase (AST), total cholesterol (TC), triglycerides, high-density lipoprotein cholesterol (HDL-C), low-density lipoprotein cholesterol (LDL-C), and fasting plasma glucose (FPG). Third, renal function and clinical history included estimated glomerular filtration rate (eGFR), which was calculated using the CKD-EPI equation adapted for the Chinese population (34, 35), as well as disease history, including diabetes, hypertension, and hyperlipidemia, and medication use, including antiplatelet agents, lipid-lowering drugs, diuretics, beta-blockers, calcium channel blockers, and angiotensin-converting enzyme inhibitors (ACEIs)/angiotensin receptor blockers (ARBs). Detailed definitions of selected diseases are provided in the **Supplementary Materials**.

### 2.3 Calculation of inflammatory markers

Calculate the four inflammatory markers using the following formulas (28, 30, 32, 33): AISI = (Neutrophil count × Monocyte count × Platelet count)/Lymphocyte count; SIRI = (Neutrophil count × Monocyte count)/Lymphocyte count; SII = (Platelet count × Neutrophil count)/Lymphocyte count; PLR = Platelet count/Lymphocyte count.

### 2.4 Outcome

The primary endpoint of this study was the development of new-onset CKD during follow-up. CKD was diagnosed according to the KDIGO guidelines and was defined as a persistently reduced eGFR of <60 mL/min/1.73 m², calculated using the CKD-EPI equation, and/or a persistently elevated urinary albumin-to-creatinine ratio of >30 mg/g (2, 36, 37). To confirm chronicity, at least two consecutive measurements meeting either of these criteria and obtained more than 3 months apart were required, and cases of acute kidney injury were excluded. The date of the first laboratory test meeting the diagnostic criteria was recorded as the time of endpoint occurrence.

### 2.5 Statistical analysis

Participants were classified into CKD and non-CKD groups according to study outcome, and their baseline demographic and clinical characteristics were compared between the two groups. To evaluate the association between inflammatory markers and CKD risk, AISI, SIRI, SII, and PLR were categorized into tertiles. Before conducting Cox regression analysis, the proportional hazards assumption was tested and confirmed to be satisfied (**Supplementary Figure 1**). Cox regression models were then used to examine these associations, with multivariable models constructed through progressive adjustment for potential confounders. In addition, cumulative risk curves (CRC) were plotted to visually compare CKD risk across tertile groups.

Restricted cubic splines (RCS) were further applied to explore the dose-response relationships between each inflammatory marker and CKD risk, identify potential inflection points, and perform two-segment comparison analyses. To compare the predictive performance of the different inflammatory markers, receiver operating characteristic (ROC) curves, the C-index, and random forest (RF) variable importance rankings were used for a comprehensive evaluation.

Statistical significance was defined as a two-sided P value <0.05. All analyses were performed using R software (version 4.3.2).

## 3 Results

### 3.1 Basic characteristics of the study population

A total of 5181 patients with CAD from three centers were included in this study. During a median follow-up of 4.85 years, 737 patients (14.23%) developed CKD. The baseline characteristics of the CKD and non-CKD groups are presented in **Table 1**.

**Table 1:** Baseline characteristics of CKD and non-CKD patients.

Characteristic	Overall	Non-CKD	CKD	P value
Number	5181	4444	737	
Age (years)	58.48±8.28	58.59±8.32	57.86±7.99	0.028
Sex (%)				<0.001
Male	3718 (71.76%)	3126 (70.34%)	592 (80.33%)	
Female	1463 (28.24%)	1318 (29.66%)	145 (19.67%)	
BMI (kg/m^2^)	25.76±3.97	25.67±3.98	26.30±3.93	<0.001
SBP (mmHg)	143.54±17.87	141.95±17.76	145.61±17.94	<0.001
DBP (mmHg)	88.14±13.52	87.80±13.35	89.76±14.56	<0.001
Smoking (%)	701 (13.53%)	555 (12.49%)	146 (19.81%)	<0.001
Drinking (%)	514 (9.92%)	393 (8.84%)	121 (16.42%)	<0.001
Laboratory tests				
ALT (U/L)	17.00 (12.00-28.00)	17.00 (12.00-27.20)	20.90 (13.46-31.00)	<0.001
AST (U/L)	18.30 (15.00-24.00)	18.00 (15.00-24.00)	19.30 (16.00-26.00)	<0.001
TC (mmol/L)	4.10±0.92	4.09±0.91	4.17±0.98	0.025
TG (mmol/L)	0.65 (0.55-1.46)	0.65 (0.55-1.37)	0.69 (0.58-1.97)	<0.001
HDL-C (mg/dL)	2.97±0.76	3.14±0.75	2.67±0.79	<0.001
LDL-C (mg/dL)	1.12±0.28	1.10±0.29	1.18±0.28	<0.001
FPG (mmol/L)	4.75±0.90	4.72±0.92	4.90±0.76	<0.001
eGFR (ml/min/1.73 m^2^)	114.69±22.01	115.79±23.00	105.98±14.24	<0.001
AISI	116.10 (80.74-161.38)	110.29 (78.30-152.61)	171.80 (111.98-265.95)	<0.001
SIRI	0.95 (0.75-1.22)	0.92 (0.73-1.16)	1.18 (0.88-1.60)	<0.001
SII	453.35 (328.74-611.84)	435.04 (319.56-579.10)	609.10 (421.98-824.85)	<0.001
PLR	130.80±47.33	127.87±45.27	148.47±55.10	<0.001
Medical history				
Diabetes (%)	351 (6.76%)	290 (6.53%)	61 (8.25%)	<0.001
Hypertension (%)	2899 (55.95%)	2162 (48.65%)	737 (100.00%)	<0.001
Dyslipidemia (%)	2430 (46.90%)	1969 (44.31%)	461 (62.55%)	<0.001
Medications				
Lipid-lowering drugs (%)	5153 (99.46%)	4419 (99.44%)	734 (99.59%)	0.594
antiplatelet drugs (%)	5124 (98.90%)	4393 (98.85%)	731 (99.19%)	0.421
Diuretics (%)	956 (18.45%)	766 (17.24%)	190 (25.77%)	<0.001
Beta-blockers (%)	5032 (97.12%)	4303 (96.83%)	729 (98.91%)	0.002
ACEIs/ARBs (%)	4647 (89.69%)	3942 (88.70%)	705 (95.66%)	<0.001

Data are presented as mean ± standard deviation, median (interquartile range), or as numbers, and percentages.

CKD, chronic kidney disease; BMI, body mass index; SBP, systolic blood pressure; DBP, diastolic blood pressure; ALT, alanine transaminase; AST, aspartate transaminase; HDL-C, high-density lipoprotein cholesterol; LDL-C, low-density lipoprotein cholesterol; TC, total cholesterol; TG, triglyceride; FPG, fasting plasma glucose; AISI, aggregate index of systemic inflammation; SII, Systemic Immune-Inflammation Index; SIRI, Systemic Inflammation Response Index; ACEIs, angiotensin-converting enzyme inhibitors; ARBs, angiotensin receptor blockers.

Compared with the non-CKD group, patients who developed CKD showed a less favorable clinical profile. In terms of demographic and lifestyle characteristics, the CKD group had a higher proportion of men and a younger age, along with significantly higher BMI, SBP, and DBP, as well as higher rates of smoking and alcohol consumption. Regarding laboratory findings, patients in the CKD group had significantly higher levels of transaminases, TC, triglycerides, LDL-C, and FPG, whereas HDL-C and eGFR were significantly lower. In addition, inflammatory marker levels, including AISI, SIRI, SII, and PLR, were all significantly higher in the CKD group.

With respect to comorbidities and medication use, patients in the CKD group had significantly higher prevalences of diabetes, hypertension, and dyslipidemia. They were also more likely to use diuretics, beta-blockers, and ACEIs/ARBs. Furthermore, baseline characteristics were additionally compared across the five CKD stages, and the results were generally consistent with the findings described above (**Supplementary Table 1**).

### 3.2 Association between inflammatory markers and CKD risk in CAD patients

**Figure 2:**
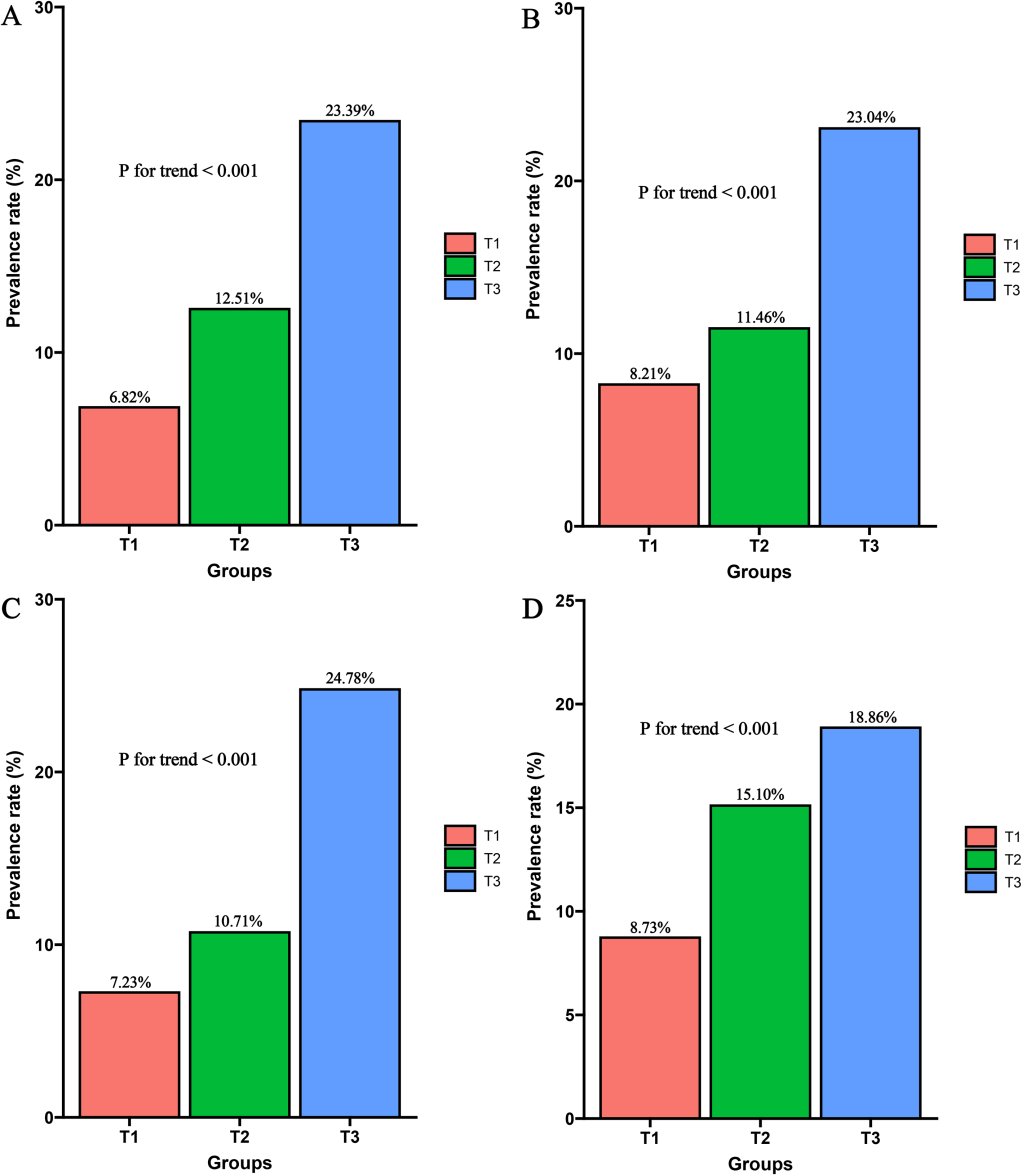
Incidence of CKD across different groups. **(A)** AISI; **(B)** SIRI; **(C)** SII; **(D)** PLR. T1: The first tertile group; T2: The second tertile group; T3: The third tertile group. Four groups were compared using the chi-square test for trend.

The four inflammatory markers were first divided into tertiles (T1–T3). The analysis showed that the incidence of CKD increased progressively with higher levels of these markers, with the T3 group showing a markedly higher incidence than the T2 and T1 groups (**Figure 2**).

**Figure 3:**
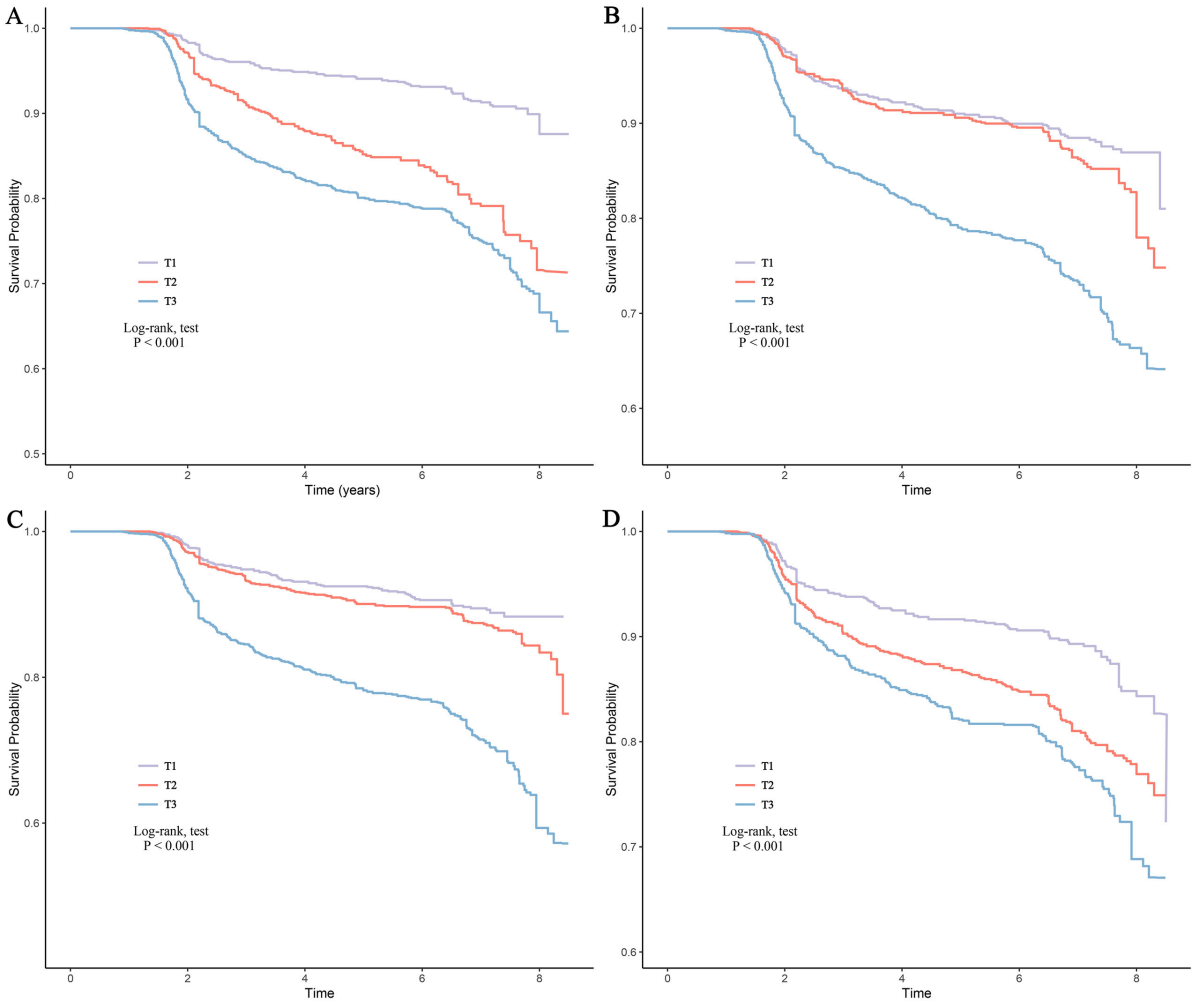
Cumulative risk curves of CKD across different groups. **(A)** AISI; **(B)** SIRI; **(C)** SII; **(D)** PLR. T1: The first tertile group; T2: The second tertile group; T3: The third tertile group.

In multivariable Cox regression analyses, elevated levels of all four inflammatory markers were independently associated with an increased risk of CKD. In Model 5, each standard deviation increase in AISI, SIRI, SII, and PLR was associated with a 2.773-fold [hazard ratio (HR): 2.773, 95% confidence interval (CI): 2.523–3.048], 2.560-fold (HR: 2.560, 95% CI: 2.221–2.951), 2.052-fold (HR: 2.052, 95% CI: 1.846–2.281), and 2.095-fold (HR: 2.095, 95% CI: 1.877–2.366) higher risk of CKD, respectively (**Table 2**). When the markers were analyzed by tertiles, a clear graded dose-response relationship was observed. Compared with T1, both T2 and T3 were associated with significantly higher CKD risk, and the risk increased progressively from T1 to T3 (**Table 2**). These findings were further supported by the Kaplan–Meier CRC (**Figure 3**), which showed that patients in T2 and T3 had significantly higher cumulative CKD incidence than those in T1.

**Table 2:** Relationship between inflammatory markers and CKD risk in patients with CAD.

CKD	Model 1	Model 2	Model 3	Model 4	Model 5
	HR (95% CI) P	HR (95% CI) P	HR (95% CI) P	HR (95% CI) P	HR (95% CI) P
AISI					
AISI (per 1SD increase)	3.400 [3.153, 3.665] <0.001	3.121 [2.884, 3.377] <0.001	3.131 [2.880, 3.404] <0.001	3.053 [2.788, 3.342] <0.001	2.773 [2.523, 3.048] <0.001
Tertiles of AISI					
Tertile 1	Reference	Reference	Reference	Reference	Reference
Tertile 2	3.454 [2.706, 4.407] <0.001	2.650 [2.076, 3.383] <0.001	2.271 [1.812, 2.846] <0.001	2.132 [1.701, 2.672] <0.001	1.906 [1.518, 2.393] <0.001
Tertile 3	5.063 [4.039, 6.347] <0.001	4.861 [3.861, 6.120] <0.001	3.445 [2.805, 4.230] <0.001	3.434 [2.794, 4.221] <0.001	3.149 [2.560, 3.873] <0.001
P for trend	<0.001	<0.001	<0.001	<0.001	<0.001
SIRI					
SIRI (per 1SD increase)	3.884 [3.413, 4.420] <0.001	3.693 [3.222, 4.233] <0.001	3.629 [3.184, 4.137] <0.001	2.946 [2.557, 3.394] <0.001	2.560 [2.221, 2.951] <0.001
Tertiles of SIRI					
Tertile 1	Reference	Reference	Reference	Reference	Reference
Tertile 2	1.421 [1.141, 1.771] 0.002	1.374 [1.099, 1.718] 0.005	1.290 [1.040, 1.600] 0.021	1.208 [0.972, 1.500] 0.088	1.185 [0.953, 1.473] 0.127
Tertile 3	2.388 [1.946, 2.930] <0.001	2.328 [1.896, 2.859] <0.001	2.680 [2.213, 3.247] <0.001	2.664 [2.197, 3.230] <0.001	2.483 [2.046, 3.014] <0.001
P for trend	<0.001	<0.001	<0.001	<0.001	<0.001
SII					
SII (per 1SD increase)	2.592 [2.353, 2.857] <0.001	2.584 [2.358, 2.831] <0.001	2.490 [2.269, 2.732] <0.001	2.069 [1.869, 2.291] <0.001	2.052 [1.846, 2.281] <0.001
Tertiles of SII					
Tertile 1	Reference	Reference	Reference	Reference	Reference
Tertile 2	1.604 [1.269, 2.027] <0.001	1.511 [1.193, 1.914] <0.001	1.347 [1.070, 1.695] 0.011	1.316 [1.048, 1.653] 0.018	1.313 [1.047, 1.648] 0.019
Tertile 3	2.988 [2.419, 3.690] <0.001	2.822 [2.279, 3.496] <0.001	3.365 [2.753, 4.113] <0.001	3.365 [2.753, 4.113] <0.001	3.364 [2.755, 4.107] <0.001
P for trend	<0.001	<0.001	<0.001	<0.001	<0.001
PLR					
PLR (per 1SD increase)	2.515 [2.122, 2.980] <0.001	2.394 [2.038, 2.813] <0.001	2.368 [2.011, 2.787] <0.001	2.257 [1.926, 2.569] <0.001	2.095 [1.877, 2.366] <0.001
Tertiles of PLR					
Tertile 1	Reference	Reference	Reference	Reference	Reference
Tertile 2	1.682 [1.373, 2.061] <0.001	1.596 [1.305, 1.951] <0.001	1.577 [1.290, 1.927] <0.001	1.408 [1.155, 1.718] <0.001	1.285 [1.128, 1.721] <0.001
Tertile 3	2.531 [2.058, 3.112] <0.001	2.431 [1.947, 2.893] <0.001	2.065 [1.700, 2.508] <0.001	2.054 [1.692, 2.493] <0.001	2.057 [1.696, 2.496] <0.001
P for trend	<0.001	<0.001	<0.001	<0.001	<0.001

Model 1: no covariates were adjusted. .

Model 2: age, sex, BMI, smoking status and drinking status were adjusted.

Model 3: Model 2 plus adjustment for SBP, DBP, TC, TG, HDL.C, LDL.C, and FPG.

Model 4: Model 3 plus adjustment for Diabetes, Dyslipidemia and Hypertension.

Model 5: Model 4 plus adjustment for use of antiplatelet drugs, Lipid-lowering drugs, diuretics, beta-blockers, calcium channel blockers, and ACEIs/ARBs.

CKD, chronic kidney disease; CAD, coronary artery disease; AISI, aggregate index of systemic inflammation; SIRI, Systemic Inflammation Response Index; SII, Systemic Immune-Inflammation Index; PLR, platelet-to-lymphocyte ratio; HR, hazard ratio; CI, confidence interval

Other abbreviations, see **Table 1**.

To further account for the potential influence of sex differences, stratified analyses were performed separately in men and women. The results remained consistent across both sexes, showing that higher levels of all four inflammatory markers were significantly associated with an increased risk of CKD in patients with CAD (**Supplementary Table 2**). In addition, E-value analysis was conducted to assess the possible impact of unmeasured confounding. The results suggested that unmeasured confounding was unlikely to substantially explain the observed associations (**Supplementary Table 3**).

### 3.3 Dose-response analysis linking four inflammatory markers to CKD risk in CAD patients

**Figure 4:**
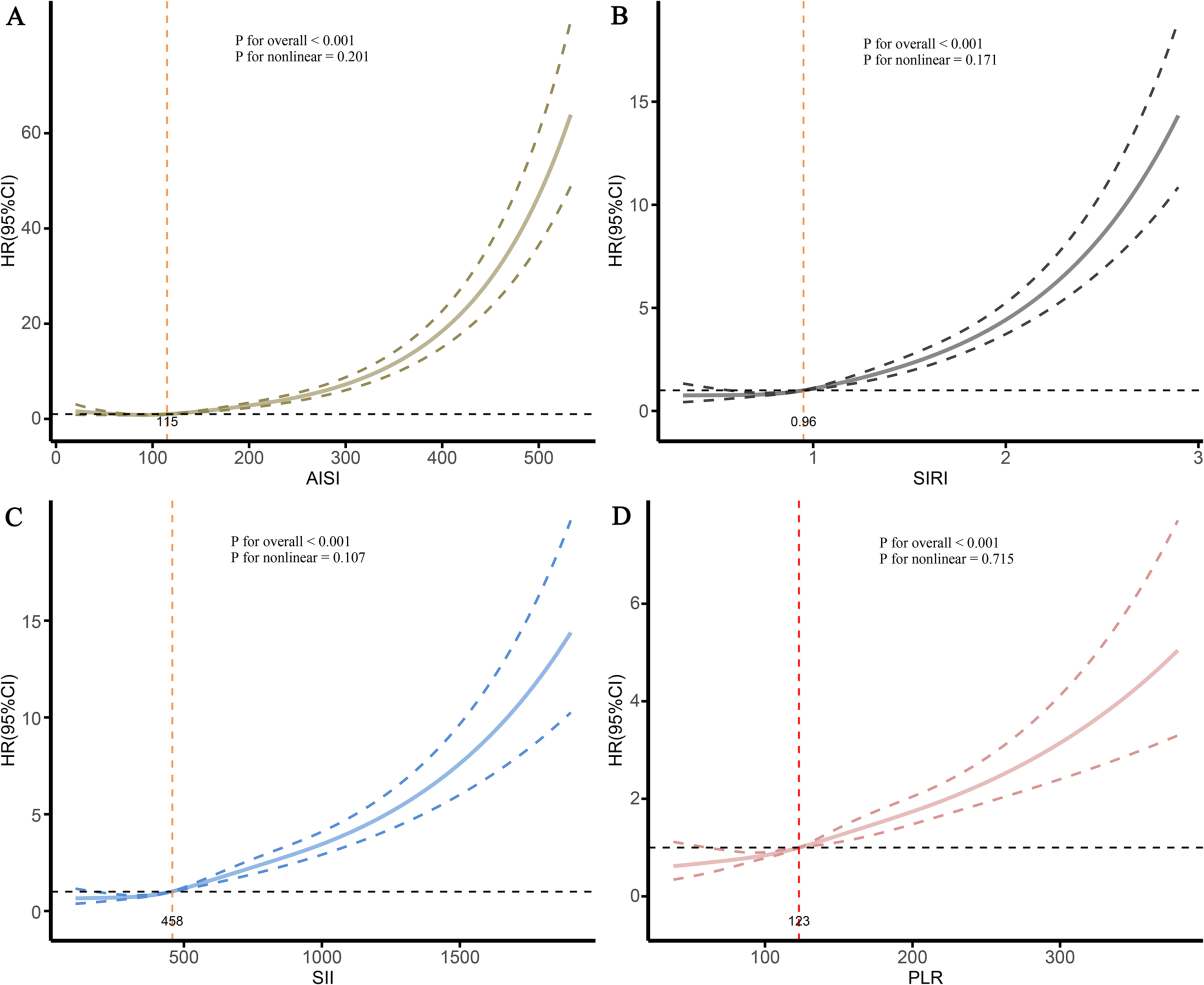
Dose-response relationship between four inflammatory markers and the risk of CKD in patients with CAD. **(A)** AISI; **(B)** SIRI; **(C)** SII; **(D)** PLR. The solid line represents the fitting line, while the dotted line represents the confidence interval.

To further investigate the shape of the associations between the four inflammatory markers and CKD risk, RCS analysis was performed. The results showed significant and progressively increasing dose-response relationships for all four markers (**Figure 4**). Inflection points indicating increased risk were identified at AISI >115, SIRI >0.96, SII >458, and PLR >123. Beyond these thresholds, the risk of CKD increased substantially (**Figure 4**). Based on these inflection points, two-piecewise Cox regression models were constructed to compare CKD risk below and above each threshold. The analysis showed that individuals with marker levels above the corresponding thresholds had significantly higher risks of CKD than those with levels at or below the thresholds (**Table 3**). The HRs were 2.437 (95% CI: 2.064–2.877) for AISI, 2.042 (95% CI: 1.730–2.411) for SIRI, 1.906 (95% CI: 1.618–2.245) for SII, and 1.243 (95% CI: 1.063–1.453) for PLR (**Table 3**).

**Table 3:** Relationship between inflammatory markers and the risk of CKD in patients with CAD based on the turning point.

CKD	Model 1	Model 2	Model 3	Model 4	Model 5
	HR (95% CI) P	HR (95% CI) P	HR (95% CI) P	HR (95% CI) P	HR (95% CI) P
AISI					
Turning point	115	115	115	115	115
<= 115	Reference	Reference	Reference	Reference	Reference
> 115	3.234 [2.698, 3.878] <0.001	2.949 [2.456, 3.540] <0.001	2.713 [2.299, 3.201] <0.001	2.687 [2.280, 3.167] <0.001	2.437 [2.064, 2.877] <0.001
SIRI					
Turning point	0.96	0.96	0.96	0.96	0.96
<= 0.96	Reference	Reference	Reference	Reference	Reference
> 0.96	2.443 [2.086, 2.861] <0.001	2.396 [2.042, 2.811] <0.001	2.365 [2.021, 2.766] <0.001	2.046 [1.736, 2.412] <0.001	2.042 [1.730, 2.411] <0.001
SII					
Turning point	458	458	458	458	458
<= 458	Reference	Reference	Reference	Reference	Reference
> 458	2.389 [2.038, 2.800] <0.001	2.389 [2.041, 2.798] <0.001	2.378 [2.032, 2.781] <0.001	2.022 [1.716, 2.382] <0.001	1.906 [1.618, 2.245] <0.001
PLR					
Turning point	123	123	123	123	123
<= 123	Reference	Reference	Reference	Reference	Reference
> 123	1.717 [1.475, 2.000] <0.001	1.711 [1.471, 1.990] <0.001	1.687 [1.450, 1.964] <0.001	1.264 [1.082, 1.476] 0.003	1.243 [1.063, 1.453] 0.006

Model 1: no covariates were adjusted. .

Model 2: age, sex, BMI, smoking status and drinking status were adjusted.

Model 3: Model 2 plus adjustment for SBP, DBP, TC, TG, HDL.C, LDL.C, and FPG.

Model 4: Model 3 plus adjustment for Diabetes, Dyslipidemia and Hypertension.

Model 5: Model 4 plus adjustment for use of antiplatelet drugs, Lipid-lowering drugs, diuretics, beta-blockers, calcium channel blockers, and ACEIs/ARBs.

CKD, chronic kidney disease; CAD, coronary artery disease; AISI, aggregate index of systemic inflammation; SIRI, Systemic Inflammation Response Index; SII, Systemic Immune-Inflammation Index; PLR, platelet-to-lymphocyte ratio; HR, hazard ratio; CI, confidence interval

Other abbreviations, see **Table 1**.

### 3.4 Comparative predictive ability of four inflammatory markers for CKD in CAD patients

**Figure 5:**
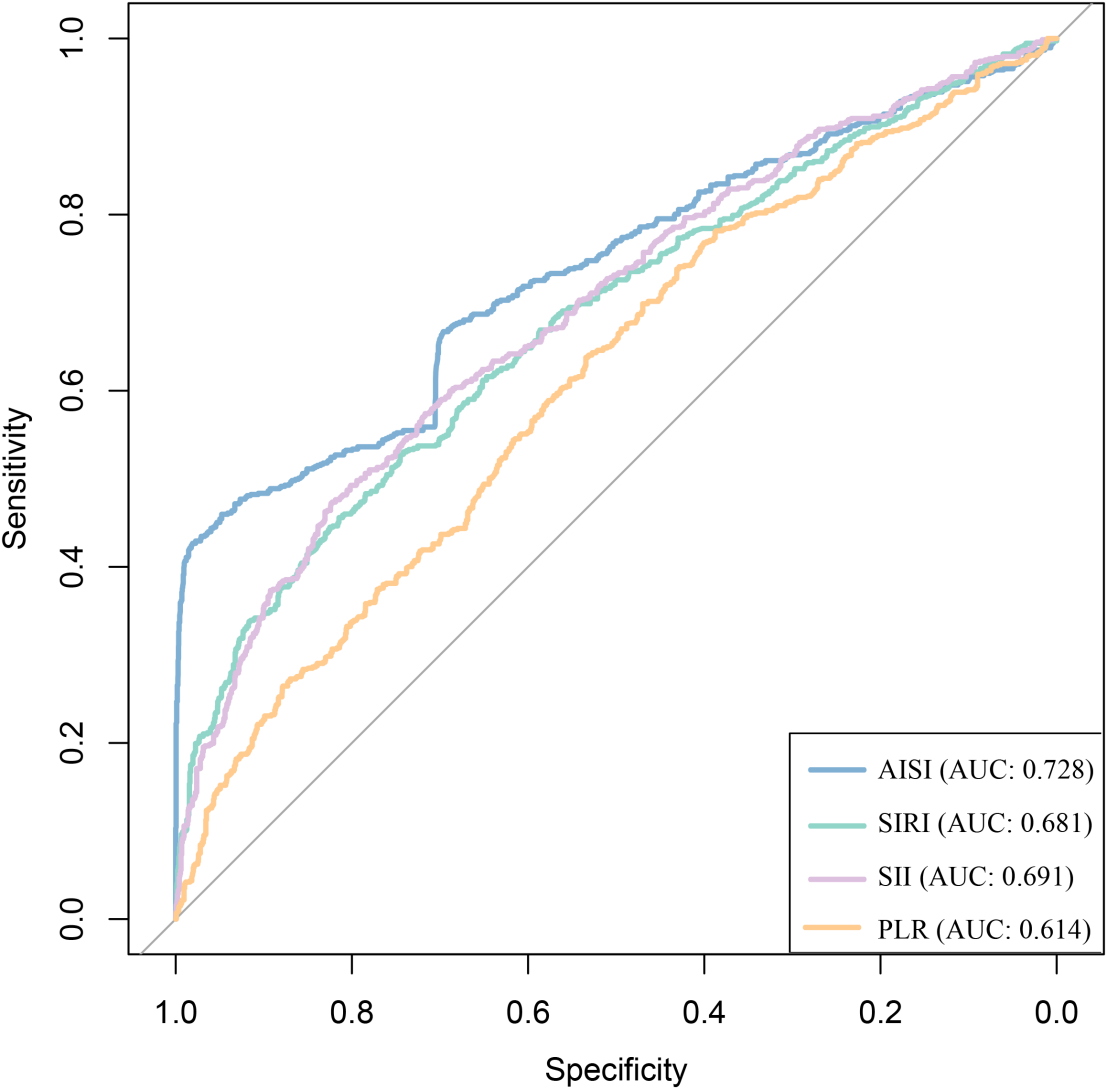
Compare the predictive ability of four inflammatory markers for the risk of CKD in patients with CAD.

**Figure 6:**
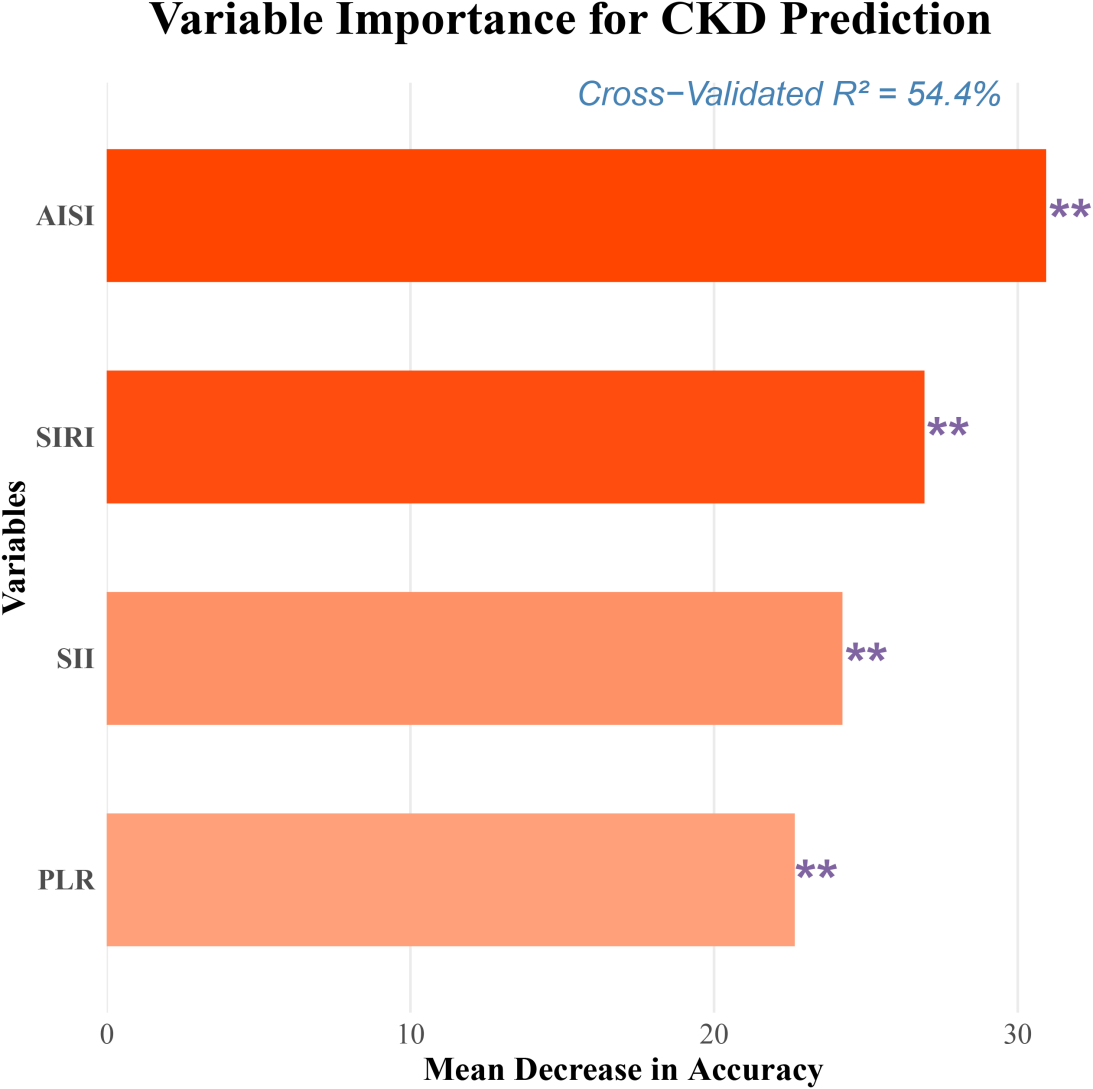
Comparison of the variable importance of four inflammatory markers under the random forest model. **: P < 0.01.

To evaluate and compare the predictive performance of the four inflammatory markers for CKD risk, an integrated analysis was conducted. ROC curve analysis showed that all four markers had predictive value, with area under the curve (AUC) values of 0.728 for AISI, 0.681 for SIRI, 0.691 for SII, and 0.614 for PLR (**Table 4**, **Figure 5**). Among them, AISI had a significantly higher AUC than the other three markers, indicating superior discriminative ability (**Table 4**, **Figure 5**). To further assess incremental predictive value, each inflammatory marker was added separately to the fully adjusted Model 5, and the C-index was calculated. The model including AISI achieved the highest C-index (0.931), representing an increase of 0.039 compared with Model 5 alone, suggesting that AISI provided the greatest incremental predictive value (**Table 5**). In addition, variable importance ranking based on the RF algorithm consistently identified AISI as the most important predictor among the four inflammatory markers (**Figure 6**).

**Table 4:** Comparison of predictive performance of different inflammatory biomarkers for CKD risk in patients with CAD.

Inflammatory markers	AUC	95%CI low	95%CI up	Specificity	Sensitivity	Positive-pv	Negative-pv
Stroke							
AISI	0.728	0.706	0.751	0.944	0.445	0.566	0.911
SIRI	0.681	0.658	0.703	0.741	0.531	0.254	0.905
SII	0.691	0.669	0.713	0.822	0.474	0.306	0.904
PLR	0.614	0.592	0.636	0.534	0.638	0.185	0.899

CKD, chronic kidney disease; CAD, coronary artery disease; AISI, aggregate index of systemic inflammation; SIRI, Systemic Inflammation Response Index; SII, Systemic Immune-Inflammation Index; PLR, platelet-to-lymphocyte ratio; AUC, area under the curve; Positive-pv, positive predictive value; Negative-pv, negative predictive value.

Other abbreviations, see **Table 1**.

**Table 5:** Incremental predictive value of different inflammatory markers for CKD risk in CAD patients.

Inflammatory indices	C-index
Model 5	0.892
+AISI	0.931
+SIRI	0.916
+SII	0.914
+PLR	0.905

CKD, chronic kidney disease; CAD, coronary artery disease; AISI, aggregate index of systemic inflammation; SII, Systemic Immune-Inflammation Index; SIRI, Systemic Inflammation Response Index; PLR, platelet-to-lymphocyte ratio.

Other abbreviations, see **Table 1**.

Overall, the findings from ROC analysis, C-index comparison, and RF variable importance assessment consistently indicated that AISI exhibits a relatively strong predictive performance for future CKD risk among the four inflammatory markers in patients with CAD. These results suggest that AISI may serve as a reliable inflammatory indicator for CKD risk assessment in this population.

## 4 Discussion

Inflammation is a key contributor to renal impairment, and patients with CAD often exist in a chronic inflammatory state (38, 39). Therefore, monitoring inflammatory status in this population is important for assessing the risk of CKD. However, previous studies have mainly relied on single inflammatory markers, which may not adequately capture the complexity of systemic inflammation (40, 41). To address this limitation, our multicenter cohort study is the first to systematically investigate the associations between multiple novel composite inflammatory markers and subsequent CKD development in patients with CAD. The results showed that all four composite inflammatory markers—AISI, SIRI, SII, and PLR—were significantly associated with an increased risk of CKD. Threshold analysis further indicated that CKD risk increased substantially when AISI exceeded 115, SIRI exceeded 0.96, SII exceeded 458, or PLR exceeded 123. These findings suggest that maintaining these inflammatory markers below the identified thresholds may help reduce the future risk of CKD in patients with CAD. In addition, we comprehensively evaluated the predictive performance of these markers using multiple methods, including ROC analysis, the C-index, and RF–based variable importance ranking. Among them, AISI, a composite index derived easily from routine blood parameters, showed the best predictive performance and may serve as a practical clinical indicator for identifying CAD patients at high risk of CKD, thereby supporting timely intervention and risk management.

AISI, SIRI, SII, and PLR are recently developed composite indices calculated from routine complete blood cell counts (30, 42). By integrating multiple hematological parameters, these markers provide a more comprehensive and accurate reflection of systemic inflammatory activity than individual cell counts or simple ratios (31, 43). In recent years, growing evidence has demonstrated their significant predictive value across a variety of diseases (30, 31, 42, 44). For example, studies on non-alcoholic fatty liver disease have shown that several inflammatory markers are closely associated with disease presence, among which AISI exhibited the strongest predictive performance (30). In the field of bone health, SIRI has been reported to correlate significantly with the risk of bone loss and to outperform traditional single inflammatory indicators (31). In addition, a cross-sectional analysis of data from the US National Health and Nutrition Examination Survey found that higher SIRI levels were associated with a higher prevalence of CKD in the general population (42). Because patients with CAD often have a more pronounced inflammatory burden, these findings provide relevant support for the rationale of our study (45, 46). In infectious diseases such as sepsis, SIRI has also been shown to outperform single markers such as C-reactive protein and white blood cell count in assessing disease severity and predicting mortality (28). Taken together, evidence from previous studies, together with our findings, suggests that these readily available composite inflammatory markers may have broad value in risk stratification and clinical decision-making across different disease settings.

The development of CKD in patients with CAD under prolonged inflammatory conditions is a complex process involving multiple interconnected pathophysiological pathways. First, the chronic low-level inflammation represented by SIAI can lead to endothelial dysfunction and microvascular damage (47). Inflammatory mediators such as TNF-α and IL-6 inhibit nitric oxide synthesis and increase vascular permeability, thereby causing glomerular endothelial damage, proteinuria, local ischemia, and a decline in glomerular filtration rate (48–50). Second, inflammation can directly activate the RAAS, leading to increased angiotensin II production (51, 52). Angiotensin II not only promotes oxidative stress and fibrotic responses but also constricts the efferent arterioles of the glomeruli, resulting in intraglomerular hypertension and further injury to the filtration barrier (2, 53, 54). In addition, reactive oxygen species released by inflammatory cells may exceed the antioxidant capacity of the kidney, triggering oxidative stress and mitochondrial dysfunction (55–57). This process can induce lipid peroxidation, DNA damage, and apoptosis of renal tubular epithelial cells, thereby accelerating tubulointerstitial fibrosis (58, 59). Chronic inflammation may also disrupt insulin signaling pathways, thereby aggravating glucose and lipid metabolic abnormalities as well as insulin resistance (60, 61). Metabolic disturbances such as hyperglycemia and elevated free fatty acids can directly damage podocytes and renal tubules, while also promoting the accumulation of advanced glycation end-products, which further accelerates renal sclerosis (62–64). Finally, elevated inflammatory marker levels may contribute to a prothrombotic state and microcirculatory dysfunction, thereby promoting platelet activation and intrarenal microthrombosis (65–67). These changes aggravate renal ischemia and hypoxia and further damage the tubular and interstitial structures of the kidney (68, 69). Taken together, these mechanisms are closely interrelated and may act synergistically to form a vicious cycle of inflammation, endothelial injury, fibrosis, and progressive renal function loss. CKD itself is also characterized by persistent inflammation and oxidative stress, which may further worsen cardiovascular outcomes in patients with CAD (70, 71).

This study was based on a multicenter cohort design involving three medical centers in China. The relatively large sample size and inclusion of participants from different geographic regions enhanced the representativeness and generalizability of the findings. In addition, comprehensive statistical analyses were performed, which not only strengthened the reliability of the results but also allowed a systematic comparison of the predictive performance of different inflammatory markers for CKD risk in patients with CAD. The findings consistently showed that AISI had the best predictive performance among these markers, suggesting that it may serve as a simple and clinically accessible tool for inflammatory assessment. These results provide important support for renal risk stratification and early intervention in patients with CAD.

Of course, several limitations of this study should be acknowledged. First, although this was a multicenter study, all participants were recruited from China, and the cohort was predominantly male. Therefore, the generalizability of our findings to other populations, particularly those with different geographic or ethnic backgrounds, requires further validation. Second, the analysis relied solely on baseline data and did not account for dynamic changes in inflammatory markers during follow-up. Future studies should further investigate the associations between longitudinal changes in these markers and CKD risk. In addition, information on the use of anti-inflammatory medications and biologic agents during the follow-up period was unavailable, which may have influenced the observed outcomes. Future studies are therefore encouraged to systematically collect such treatment-related data. Finally, although we adjusted for multiple known confounders in the statistical analyses, residual confounding from unmeasured factors cannot be completely excluded. Nevertheless, E-value sensitivity analysis suggested that a relatively strong unmeasured confounder would be required to fully explain the main findings, which supports the robustness of our results.

## 5 Conclusion

This study demonstrates that all four composite inflammatory indices are significantly associated with the risk of CKD in patients with CAD. Among them, AISI shows a relatively strong predictive value. As an easily calculated marker, AISI may provide a more comprehensive assessment of inflammatory activity in patients with CAD. Therefore, it may be useful for the early identification of high-risk individuals, monitoring of inflammatory status, and guidance of intervention strategies. These findings also suggest that controlling inflammation may help prevent or delay the onset of CKD. Nevertheless, because this was an observational longitudinal study, caution is still needed when interpreting and generalizing the results.

## Data Availability

The raw data supporting the conclusions of this article will be made available by the authors, without undue reservation.
