# Supplementary material for: Inflammatory markers and increased risk of chronic kidney disease in patients with coronary artery disease: findings from a multicenter cohort study
Source: Front Endocrinol (Lausanne). 2026 Apr 23;17:1827326. doi: 10.3389/fendo.2026.1827326 (PMC13149072; doi:10.3389/fendo.2026.1827326)
Supplement: Supplementary file 1 [file DataSheet1.docx]

Supplementary Material

1 Supplemental material and methods

Baseline examination

Anthropometric measurements were taken by trained nurses. Data for height and weight were 3 acquired following a protocol standardized to an accuracy of 0.1 kg and 0.1 cm, respectively. Current smokers were defined as having smoked 100 cigarettes in their lifetime and currently smoking. Alcohol consumption was evaluated with questions rgarding the types of alcoholic beverages, the frequency of alcohol consumption per week, and the usual amount consumed per occasion. Subjects who reported alcohol consumption >140 g/week for men and >70 g/week for women were deemed to have excessive alcohol consumption. Blood pressure was measured using a electronic sphygmomanometer after the patient had rested quietly for at least 10 minutes, and the average of multiple measurements was taken as the systolic and diastolic blood pressure values. All biochemical tests were measured by blood sampling after an overnight fast.

Definitions

Criteria for hypertension included self-reported hypertension, current use of anti-hypertensive medication, or systolic blood pressure (SBP) ≥ 140 mmHg and/or diastolic blood pressure (DBP) ≥ 90 mmHg recorded for at least three consecutive readings. Hyperlipidemia is defined as an abnormal elevation of lipid levels in the bloodstream, primarily characterized by increased concentrations of total cholesterol (TC), low-density lipoprotein cholesterol (LDL-C), and triglycerides (TG), or a reduction in high-density lipoprotein cholesterol (HDL-C). According to international guidelines, hyperlipidemia is diagnosed when TC levels exceed 6.2 mmol/L (240 mg/dL), LDL-C levels are ≥ 4.1 mmol/L (160 mg/dL), TG levels are ≥ 2.3 mmol/L (200 mg/dL), or HDL-C levels fall below 1.0 mmol/L (40 mg/dL) in men and 1.3 mmol/L (50 mg/dL) in women. Coronary artery disease (CAD) was diagnosed primarily based on coronary angiography findings, with luminal stenosis ≥50% in the left main or major coronary branches confirming the diagnosis. Additionally, patients with a history of fatal or non‑fatal myocardial infarction, unstable angina, or prior coronary revascularization procedures (e.g., percutaneous coronary intervention or coronary artery bypass grafting) were also classified as having CAD.

**Details of the statistical analyses**.

Regarding missing data, we acknowledge that some covariates had missing values. To address this, we used multiple imputation with the Mice package in R. All covariates had a missing proportion of less than 20%. Five imputed datasets were generated, and the results were pooled to ensure that the imputed data preserved the original distribution and characteristics.

In addition, to verify the appropriateness of the Cox regression model, we tested the proportional hazards assumption for the association between the four inflammatory markers and the risk of chronic kidney disease. The results indicated that the proportional hazards assumption was satisfied.

Variables of baseline characteristics are shown as n (%) if categorical, mean (SD) if normally distributed, and median (interquartile range) if nonnormally distributed. To compare the baseline characteristics of the two different groups, the chi-square test was used for categorical variables, and independent sample T-tests or non-parametric tests were employed for continuous variables that were normally distributed or skewed.

The association between inflammatory markers and future risk of CKD in patients with coronary artery disease was tested with multivariable Cox regression models. This study set four different models (Model 1: unadjusted; Model 2: age, sex, BMI, smoking status and drinking status were adjusted; Model 3: Model 2 plus adjustment for SBP, DBP, TC, TG, HDL.C, LDL.C, and FPG. Model 4: Model 3 plus adjustment for Diabetes, Dyslipidemia and Hypertension; Model 5: Model 4 plus adjustment for use of antiplatelet drugs, Lipid-lowering drugs, diuretics, beta-blockers, calcium channel blockers, and ACEIs/ARBs) to adjust. Tests for trend were conducted, assigning the median value within each tertile to the corresponding tertile. In addition, the restricted inverse square spline (four nodes at the 5th, 35th, 65th, and 95th percentiles of the inflammatory markers distribution) was used to evaluate the nonlinear relationships. Finally, to compare the predictive utility of the different inflammatory markers, receiver operating characteristic (ROC) curves, the C-index, and random forest (RF) variable importance rankings were applied for comprehensive evaluation.

All analyses were done using R (version 4.3.2). All P-values were two-sided, and P-values of <0.05 denoted statistical significance.

**2 Supplementary Tables**

**Table S1.** Baseline characteristics based on the CKD classification into 5 stages

| Characteristic | Non-CKD | CKD-1 | CKD-2 | CKD-3 | CKD-4 | CKD-5 | P value |
| --- | --- | --- | --- | --- | --- | --- | --- |
| N | 3288 | 387 | 769 | 657 | 61 | 19 |  |
| Age (years) | 58.68±8.24 | 58.80±8.47 | 58.09±8.58 | 58.01±7.93 | 56.54±8.28 | 57.00±8.93 | 0.070 |
| Sex (%) |  |  |  |  |  |  | <0.001 |
| Male | 2316 (70.44%) | 273 (70.54%) | 537 (69.83%) | 529 (80.52%) | 50 (81.97%) | 13 (68.42%) |  |
| Female | 972 (29.56%) | 114 (29.46%) | 232 (30.17%) | 128 (19.48%) | 11 (18.03%) | 6 (31.58%) |  |
| BMI (kg/m^2^) | 25.73±4.01 | 25.53±4.14 | 25.46±3.72 | 26.23±3.98 | 26.91±3.18 | 26.60±3.64 | <0.001 |
| SBP (mmHg) | 142.29±17.32 | 145.15±17.78 | 145.18±18.28 | 145.47±19.10 | 146.84±20.28 | 146.16±19.26 | <0.001 |
| DBP (mmHg) | 87.62±13.10 | 88.06±14.70 | 88.44±13.67 | 88.63±14.25 | 89.15±16.63 | 91.89±16.91 | <0.001 |
| Smoking (%) | 407 (12.38%) | 50 (12.92%) | 98 (12.74%) | 122 (18.57%) | 17 (27.87%) | 7 (36.84%) | <0.001 |
| Drinking (%) | 302 (9.18%) | 43 (11.11%) | 48 (6.24%) | 104 (15.83%) | 11 (18.03%) | 6 (31.58%) | <0.001 |
| ALT (U/L) | 20.31±12.85 | 22.66±16.64 | 23.28±17.17 | 24.62±17.82 | 25.68±17.59 | 26.72±18.81 | <0.001 |
| AST (U/L) | 19.86±7.54 | 20.79±8.46 | 21.12±9.3 | 21.97±9.32 | 22.01±9.39 | 22.60±9.46 | <0.001 |
| TC (mmol/L) | 3.85±0.95 | 3.99±0.82 | 4.10±0.85 | 4.18±0.97 | 4.20±1.00 | 4.11±0.94 | 0.004 |
| TG (mmol/L) | 0.96±0.73 | 1.11±0.91 | 1.12±0.91 | 1.01±0.70 | 1.34±1.20 | 1.36±1.11 | <0.001 |
| HDL-C (mg/dL) | 3.15±0.76 | 3.12±0.61 | 3.02±0.89 | 2.70±0.78 | 2.66±0.78 | 2.68±0.85 | <0.001 |
| LDL-C (mg/dL) | 1.09±0.22 | 1.10±0.29 | 1.11±0.32 | 1.18±0.27 | 1.20±0.28 | 1.18±0.28 | <0.001 |
| FPG (mmol/L) | 4.66±0.85 | 4.66±0.85 | 4.76±0.91 | 4.83±0.57 | 4.90±0.79 | 4.87±0.44 | <0.001 |
| eGFR (ml/min/1.73 m^2^) | 115.80±13.51 | 115.15±13.57 | 116.06±21.72 | 108.51±19.12 | 107.19±18.90 | 105.80±21.38 | <0.001 |
| AISI | 99.79±36.62 | 148.10±35.27 | 173.35±50.76 | 171.86±83.06 | 389.69±64.47 | 463.14±106.23 | <0.001 |
| SIRI | 0.91±0.31 | 1.09±0.37 | 1.21±0.36 | 1.21±0.48 | 2.05±0.55 | 2.03±0.54 | <0.001 |
| SII | 430.29±188.25 | 529.05±205.73 | 609.22±251.47 | 623.30±300.40 | 1050.61±396.94 | 1211.16±442.82 | <0.001 |
| PLR | 124.67±43.72 | 130.55±44.78 | 140.18±49.64 | 145.43±53.52 | 169.13±59.29 | 187.18±68.41 | <0.001 |
| Hypertension (%) | 1535 (46.68%) | 205 (52.97%) | 422 (54.88%) | 657 (100.00%) | 61 (100.00%) | 19 (100.00%) | <0.001 |
| Lipid-lowering drugs (%) | 3272 (99.51%) | 385 (99.48%) | 762 (99.09%) | 656 (99.85%) | 60 (98.36%) | 18 (94.74%) | 0.021 |
| antiplatelet drugs (%) | 3259 (99.12%) | 382 (98.71%) | 752 (97.79%) | 655 (99.70%) | 59 (96.72%) | 17 (89.47%) | <0.001 |
| Diuretics (%) | 587 (17.85%) | 74 (19.12%) | 105 (13.65%) | 366 (55.71%) | 34 (55.74%) | 11 (57.89%) | <0.001 |
| Beta-blockers (%) | 3216 (97.81%) | 380 (98.19%) | 707 (91.94%) | 650 (98.93%) | 61 (100.00%) | 18 (94.74%) | <0.001 |
| ACEIs/ARBs (%) | 2955 (89.87%) | 326 (84.24%) | 661 (85.96%) | 631 (96.04%) | 58 (95.08%) | 16 (84.21%) | <0.001 |

**Abbreviations:** CKD, chronic kidney disease; BMI, body mass index; SBP, systolic blood pressure; DBP, diastolic blood pressure; ALT, alanine transaminase; AST, aspartate transaminase; HDL-C, high-density lipoprotein cholesterol; LDL-C, low-density lipoprotein cholesterol; TC, total cholesterol; TG, triglyceride; FPG, fasting plasma glucose; AISI, aggregate index of systemic inflammation; SII, Systemic Immune-Inflammation Index; SIRI, Systemic Inflammation Response Index; ACEIs, angiotensin-converting enzyme inhibitors; ARBs, angiotensin receptor blockers.

Table S2. Relationship between inflammatory markers and the risk of CKD in patients with CAD after gender stratification

| **CKD** | Model 1 | Model 2 | Model 3 | Model 4 | Model 5 |
| --- | --- | --- | --- | --- | --- |
|  | HR (95% CI) P | HR (95% CI) P | HR (95% CI) P | HR (95% CI) P | HR (95% CI) P |
| **Male** |  |  |  |  |  |
| **AISI** |  |  |  |  |  |
| AISI (per 1SD increase) | 3.100 [2.850, 3.371] <0.001 | 2.734 [2.478, 3.016] <0.001 | 2.701 [2.462, 2.962] <0.001 | 2.676 [2.448, 2.924] <0.001 | 2.559 [2.310, 2.834] <0.001 |
| Tertiles of AISI |  |  |  |  |  |
| Tertile 1 | Reference | Reference | Reference | Reference | Reference |
| Tertile 2 | 2.933 [2.262, 3.803] <0.001 | 2.608 [2.014, 3.377] <0.001 | 1.942 [1.522, 2.478] <0.001 | 1.892 [1.482, 2.415] <0.001 | 1.708 [1.336, 2.184] <0.001 |
| Tertile 3 | 5.053 [3.948, 6.467] <0.001 | 4.767 [3.744, 6.071] <0.001 | 3.034 [2.427, 3.794] <0.001 | 2.941 [2.355, 3.672] <0.001 | 2.915 [2.331, 3.645] <0.001 |
| P for trend | <0.001 | <0.001 | <0.001 | <0.001 | <0.001 |
| **SIRI** |  |  |  |  |  |
| SIRI (per 1SD increase) | 3.386 [2.939, 3.901] <0.001 | 3.182 [2.754, 3.676] <0.001 | 3.103 [2.695, 3.572] <0.001 | 2.741 [2.361, 3.184] <0.001 | 2.451 [2.106, 2.852] <0.001 |
| Tertiles of SII |  |  |  |  |  |
| Tertile 1 | Reference | Reference | Reference | Reference | Reference |
| Tertile 2 | 1.575 [1.240, 2.001] <0.001 | 1.352 [1.065, 1.717] 0.013 | 1.269 [1.004, 1.606] 0.047 | 1.239 [0.977, 1.572] 0.077 | 1.179 [0.929, 1.495] 0.176 |
| Tertile 3 | 2.540 [2.055, 3.139] <0.001 | 2.503 [2.028, 3.089] <0.001 | 2.487 [2.010, 3.077] <0.001 | 2.452 [1.964, 3.062] <0.001 | 2.399 [1.921, 2.995]<0.001 |
| P for trend | <0.001 | <0.001 | <0.001 | <0.001 | <0.001 |
| **SII** |  |  |  |  |  |
| SII (per 1SD increase) | 2.713 [2.423, 3.037] <0.001 | 2.501 [2.243, 2.788] <0.001 | 2.646 [2.364, 2.960] <0.001 | 2.214 [1.967, 2.493] <0.001 | 2.085 [1.847, 2.353] <0.001 |
| Tertiles of SII |  |  |  |  |  |
| Tertile 1 | Reference | Reference | Reference | Reference | Reference |
| Tertile 2 | 1.752 [1.354, 2.267] <0.001 | 1.744 [1.347, 2.257] <0.001 | 1.375 [1.069, 1.768] 0.013 | 1.362 [1.062, 1.747] 0.015 | 1.270 [0.988, 1.631] 0.062 |
| Tertile 3 | 3.191 [2.547, 3.999] <0.001 | 3.129 [2.501, 3.914 <0.001 | 3.069 [2.458, 3.833] <0.001 | 3.001 [2.372, 3.796] <0.001 | 2.813 [2.223, 3.561] <0.001 |
| P for trend | <0.001 | <0.001 | <0.001 | <0.001 | <0.001 |
| **PLR** |  |  |  |  |  |
| PLR (per 1SD increase) | 2.652 [2.192, 3.210] <0.001 | 2.387 [1.989, 2.865] <0.001 | 2.376 [1.984, 2.845] <0.001 | 2.279 [1.932, 2.578] <0.001 | 2.183 [1.891, 2.373] <0.001 |
| Tertiles of SIRI |  |  |  |  |  |
| Tertile 1 | Reference | Reference | Reference | Reference | Reference |
| Tertile 2 | 2.453 [1.956, 3.077] <0.001 | 1.949 [1.550, 2.451] <0.001 | 1.686 [1.345, 2.113] <0.001 | 1.544 [1.234, 1.931] <0.001 | 1.481 [1.186, 1.848] <0.001 |
| Tertile 3 | 2.426 [2.036, 3.104] <0.001 | 2.228 [1.952, 2.897] <0.001 | 2.115 [1.701, 2.629] <0.001 | 21.995 [1.607, 2.476] <0.001 | 1.937 [1.564, 2.398] <0.001 |
| P for trend | <0.001 | <0.001 | <0.001 | <0.001 | <0.001 |
| **Female** |  |  |  |  |  |
| **AISI** |  |  |  |  |  |
| AISI (per 1SD increase) | 5.624 [4.620, 6.847] <0.001 | 5.284 [4.264, 6.547] <0.001 | 5.084 [4.087, 6.325] <0.001 | 4.472 [3.556, 5.623] <0.001 | 3.912 [3.056, 5.007] <0.001 |
| Tertiles of AISI |  |  |  |  |  |
| Tertile 1 | Reference | Reference | Reference | Reference | Reference |
| Tertile 2 | 4.547 [2.463, 8.397] <0.001 | 4.299 [2.311, 7.998] <0.001 | 4.117 [2.209, 7.672] <0.001 | 4.101 [2.183, 7.549] <0.001 | 3.448 [2.806, 4.232] <0.001 |
| Tertile 3 | 7.378 [4.170, 13.054] <0.001 | 7.020 [3.995, 12.336] <0.001 | 6.076 [3.415, 10.810] <0.001 | 5.063 [4.039, 6.347] <0.001 | 4.861 [3.861, 6.120] <0.001 |
| P for trend | <0.001 | <0.001 | <0.001 | <0.001 | <0.001 |
| **SIRI** |  |  |  |  |  |
| SIRI (per 1SD increase) | 6.335 [4.665, 8.601] <0.001 | 5.727 [4.173, 7.859] <0.001 | 5.671 [4.045, 7.951] <0.001 | 4.301[3.085, 5.996] <0.001 | 3.673 [2.599, 5.190] <0.001 |
| Tertiles of SII |  |  |  |  |  |
| Tertile 1 | Reference | Reference | Reference | Reference | Reference |
| Tertile 2 | 3.555 [1.866, 6.754] <0.001 | 2.316 [1.257, 4.270] 0.007 | 1.441 [0.836, 2.484] 0.188 | 1.385 [0.800, 2.397] 0.245 | 1.284 [0.738, 2.233] 0.376 |
| Tertile 3 | 5.495 [3.000, 10.063] <0.001 | 4.880 [2.744, 8.679] <0.001 | 3.761 [2.336, 6.053] <0.001 | 3.656 [2.247, 5.950] <0.001 | 3.073 [1.874, 5.038] <0.001 |
| P for trend | <0.001 | <0.001 | <0.001 | <0.001 | <0.001 |
| **SII** |  |  |  |  |  |
| SII (per 1SD increase) | 2.512 [2.085, 3.026] <0.001 | 2.479 [2.086, 2.946] <0.001 | 2.420 [2.037, 2.876] <0.001 | 2.357 [2.013, 2.748] <0.001 | 2.163 [1.714, 2.637] <0.001 |
| Tertiles of SII |  |  |  |  |  |
| Tertile 1 | Reference | Reference | Reference | Reference | Reference |
| Tertile 2 | 2.561 [1.359, 4.828] 0.004 | 2.407 [1.301, 4.454] 0.005 | 1.229 [0.698, 2.164] 0.476 | 1.126 [0.639, 1.984] 0.682 | 1.053 [0.601, 1.846] 0.857 |
| Tertile 3 | 5.284 [3.069, 9.098] <0.001 | 5.152 [2.899, 9.156] <0.001 | 4.919 [3.089, 7.833] <0.001 | 4.507 [2.857, 7.111] <0.001 | 3.942 [2.453, 6.333] <0.001 |
| P for trend | <0.001 | <0.001 | <0.001 | <0.001 | <0.001 |
| **PLR** |  |  |  |  |  |
| PLR (per 1SD increase) | 2.311 [1.607, 3.323] <0.001 | 2.286 [1.566, 3.336] <0.001 | 2.024 [1.345, 3.046] <0.001 | 2.021 [1.343, 3.045] <0.001 | 2.015 [1.337, 3.041] <0.001 |
| Tertiles of SIRI |  |  |  |  |  |
| Tertile 1 | Reference | Reference | Reference | Reference | Reference |
| Tertile 2 | 2.008 [1.250, 3.224] 0.004 | 1.999 [1.242, 3.219] 0.004 | 1.978 [1.235, 3.169] 0.005 | 1.523 [0.903, 2.570] 0.114 | 1.500 [0.930, 2.422] 0.097 |
| Tertile 3 | 3.479 [2.096, 5.776] <0.001 | 2.574 [1.515, 4.373 <0.001 | 2.559 [1.625, 4.031] <0.001 | 2.558 [1.625, 4.026] <0.001 | 2.187 [1.379, 3.468] <0.001 |
| P for trend | <0.001 | <0.001 | <0.001 | <0.001 | <0.001 |

Model 1: no covariates were adjusted.

Model 2: age, sex, BMI, smoking status and drinking status were adjusted.

Model 3: Model 2 plus adjustment for SBP, DBP, TC, TG, HDL.C, LDL.C, and FPG.

Model 4: Model 3 plus adjustment for Diabetes, Dyslipidemia and Hypertension.

Model 5: Model 4 plus adjustment for use of antiplatelet drugs, Lipid-lowering drugs, diuretics, beta-blockers, calcium channel blockers, and ACEIs/ARBs.

Abbreviations: CKD, chronic kidney disease; CAD, coronary artery disease; AISI, aggregate index of systemic inflammation; SIRI, Systemic Inflammation Response Index; SII, Systemic Immune-Inflammation Index; PLR, platelet-to-lymphocyte ratio; HR, hazard ratio; CI, confidence interval

Other abbreviations, see Table 1.

**Table S3.** E-values for the observed associations between inflammatory markers and the risk of CKD in patients with CAD.

|  | Model 1 | Model 2 | Model 3 | Model 4 | Model 5 |
| --- | --- | --- | --- | --- | --- |
| **AISI** |  |  |  |  |  |
| AISI (per 1SD increas) | 3.400 [3.153, 3.665] <0.001 | 3.121 [2.884, 3.377] <0.001 | 3.131 [2.880, 3.404] <0.001 | 3.053 [2.788, 3.342] <0.001 | 2.773 [2.523, 3.048] <0.001 |
| E-value for point estimate | 6.257 | 5.694 | 5.714 | 5.557 | 4.991 |
| **SIRI** |  |  |  |  |  |
| SIRI (per 1SD increas) | 3.884 [3.413, 4.420] <0.001 | 3.693 [3.222, 4.233] <0.001 | 3.629 [3.184, 4.137] <0.001 | 2.946 [2.557, 3.394] <0.001 | 2.560 [2.221, 2.951] <0.001 |
| E-value for point estimate | 7.231 | 6.847 | 6.718 | 5.340 | 4.558 |
| **SII** |  |  |  |  |  |
| SII (per 1SD increas) | 2.592 [2.353, 2.857] <0.001 | 2.584 [2.358, 2.831] <0.001 | 2.490 [2.269, 2.732] <0.001 | 2.069 [1.869, 2.291] <0.001 | 2.052 [1.846, 2.281] <0.001 |
| E-value for point estimate | 4.623 | 4.607 | 4.416 | 3.556 | 3.521 |
| **PLR** |  |  |  |  |  |
| PLR (per 1SD increas) | 2.515 [2.122, 2.980] <0.001 | 2.394 [2.038, 2.813] <0.001 | 2.368 [2.011, 2.787] <0.001 | 2.257 [1.926, 2.569] <0.001 | 2.095 [1.877, 2.366] <0.001 |
| E-value for point estimate | 4.467 | 4.221 | 4.168 | 3.941 | 3.610 |

Model 1: no covariates were adjusted.

Model 2: age, sex, BMI, smoking status and drinking status were adjusted.

Model 3: Model 2 plus adjustment for SBP, DBP, TC, TG, HDL.C, LDL.C, and FPG.

Model 4: Model 3 plus adjustment for Diabetes, Dyslipidemia and Hypertension.

Model 5: Model 4 plus adjustment for use of antiplatelet drugs, Lipid-lowering drugs, diuretics, beta-blockers, calcium channel blockers, and ACEIs/ARBs.

Abbreviations: CKD, chronic kidney disease; CAD, coronary artery disease; AISI, aggregate index of systemic inflammation; SIRI, Systemic Inflammation Response Index; SII, Systemic Immune-Inflammation Index; PLR, platelet-to-lymphocyte ratio; HR, hazard ratio; CI, confidence interval

Other abbreviations, see Table 1.

**3 Supplementary Figures**


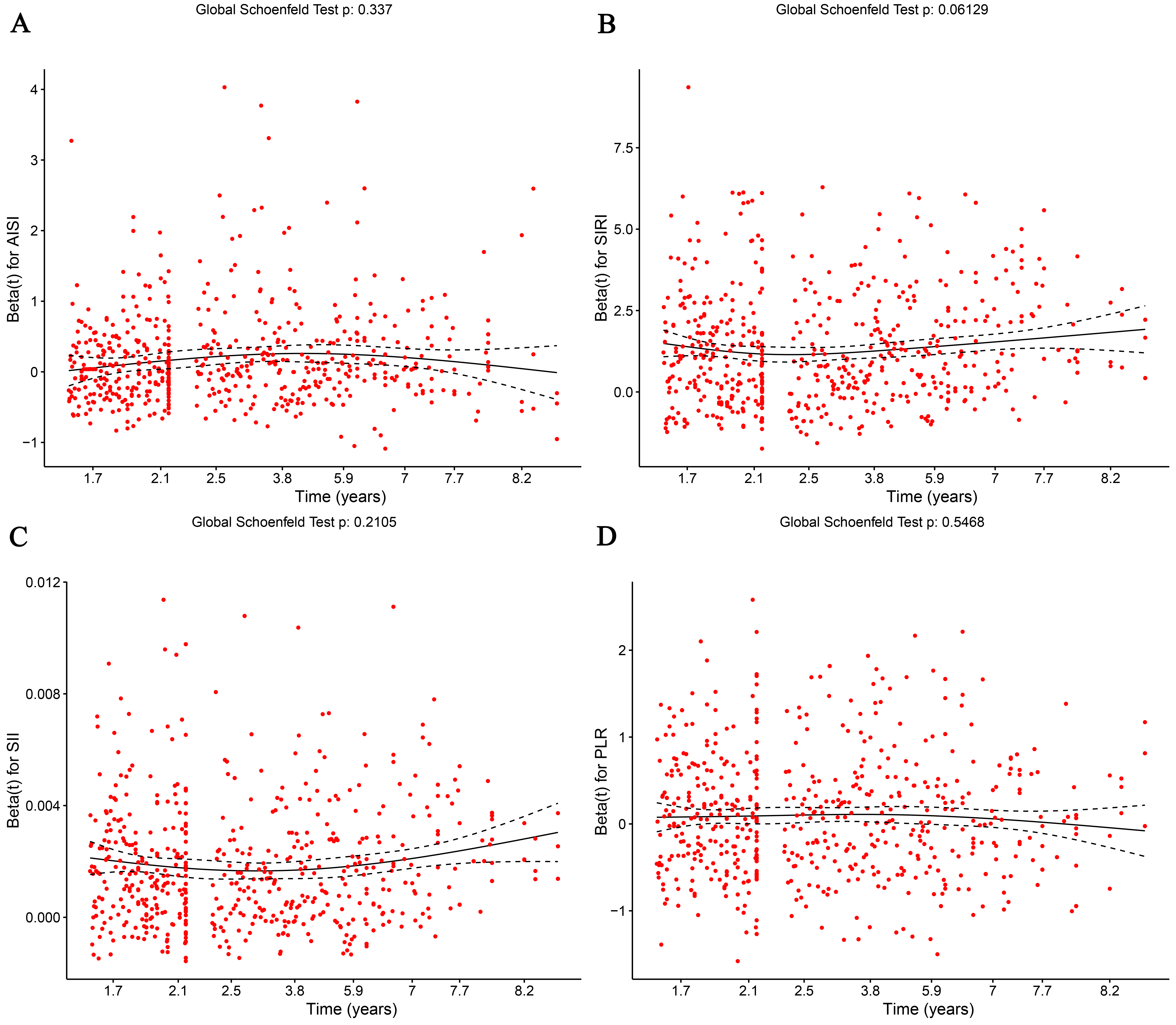


**Figure S1** Proportional hazards assumption assessment
